# Parental-reported allergic disorders and emergency department presentations for allergy in the first five years of life; a longitudinal birth cohort

**DOI:** 10.1186/s12887-018-1148-1

**Published:** 2018-05-22

**Authors:** Gerben Keijzers, Amy Sweeny, Julia Crilly, Norm Good, Cate M. Cameron, Gabor Mihala, Rani Scott, Paul A. Scuffham

**Affiliations:** 1 Department of Emergency Medicine, Gold Coast University Hospital, 1 Hospital Boulevard, Southport, QLD 4215 Australia; 2 School of Medicine, Bond University, Gold Coast, QLD Australia; 3 School of Medicine, Griffith University, Gold Coast, QLD Australia; 4 Department of Emergency Medicine, Gold Coast Health, Gold Coast, QLD Australia; 5 Menzies Health Institute, Gold Coast, QLD Australia; 6 CSIRO Digitial Productivity/ Australian e-Health Research Centre, Royal Women’s and Children’s Hospital, Brisbane, QLD Australia; 7 Jamieson Trauma Institute, Royal Brisbane & Women’s Hospital, Metro North Hospital and Health Service, Herston, QLD Australia; 8 Menzies Health Institute Queensland, Griffith University, Meadowbrook, QLD Australia; 9 Menzies Health Institute Queensland, Griffith University, Nathan, QLD Australia

**Keywords:** Allergy, Anaphylaxis, Birth cohort, Emergency department, Longitudinal study

## Abstract

**Background:**

To measure rates of parental-report of allergic disorders and ED presentations for allergic disorders in children, and to describe factors associated with either.

**Methods:**

An existing cohort of 3404 children born between 2006 and 2011 (Environments for Healthy Living) with prospectively collected pre-natal, perinatal and follow-up data were linked to i) nationwide Medicare and pharmaceutical data and ii) Emergency Department (ED) data from four hospitals in Australia. Parental-reported allergy was assessed in those who returned follow-up questionnaires. ED presentation was defined as any presentation for a suite of allergic disorders, excluding asthma. Univariate analysis and multivariate logistic regression were used to descibe risk factors for both parental-reported allergy and ED presentation for an allergic disorder.

**Results:**

The incidence of parental-reported child allergy at 1, 3 and 5 years of age was 7.8, 7.8 and 12.6%, respectively. Independent predictors of parental-report of allergy in multivariate analysis were parental-report of asthma (OR 2.2, 95% CI 1.4–3.4) or eczema (OR 4.3, 95% CI 3.1–6.1) and age > 6 months at introduction of solids (OR 1.3, 95% CI 1.0–1.7). Factors associated with ED presentations for allergy, which occurred in 3.6% of the cohort, were presence of maternal asthma (OR 2.3 95% CI:1.1, 4.9) and child born in spring (OR 1.7, 95% CI 1.1, 2.7).

**Conclusions:**

More than 10% of children up to 5 years have a parental-reported allergic disorder, and 3.6% presented to ED. Parental-report of eczema and/or asthma and late introduction of solids were predictors of parental-report of allergy. Spring birth and maternal asthma were predictors for ED presentation for allergy.

**Electronic supplementary material:**

The online version of this article (10.1186/s12887-018-1148-1) contains supplementary material, which is available to authorized users.

## Background

Allergic disorders are common and increasing, especially in children [1]. Allergic disorders consist of a wide spectrum of conditions, including rashes, atopic eczema, and most worryingly anaphylaxis. They represent an immune response to allergens, which are environmental substances that are normally considered harmless [2].

Common known triggers for allergic diseases and/or anaphylaxis include insect stings (especially from the Hymenoptera family of wasps, bees and ants), drugs (especially β-lactam antibiotics), and food (especially nuts, eggs, fish, shellfish and milk) [3]. Allergies seem to be more common in children than in adults, with food allergy prevalence reported between 7 and 10% in children [1].

The cause of the apparent increase in allergic disorders is unclear. Studies on risk factors for the development of allergic disorders have led to a number of meta-analyses of prospective cohort studies [1, 4]. From review articles [5–7] a common emerging theme is that allergic disorders are caused by a complex interrelationship between genetics, environment, and exposures both in-utero and during early infancy [5, 8]. We outline a summary of the literature on risk factors in Additional file 1.

Despite the noted high prevalence of allergic disorders in the community and the mild nature of the majority of allergic disorders, they can occasionally be more severe and anaphylactic reactions can be life-threatening. There are limited data available characterizing patients who present to the Emergency Department (ED) with allergic conditions. One French study [9] reported that allergic disorders represented 1% of all ED presentations, but was conducted nearly 20 years ago and did not report on children less than 10 years of age.

The overall aim of this study was to describe contemporaneous data for allergy presentations to the ED in the first years of life and to provide further understanding of (modifiable) associated risk factors. This study aims to measure the rates of, and describe factors associated with; 1) parental-report of allergy in children, and 2) ED presentations with allergic disorders in children in the first 5 years of life.

## Methods

### Study design

This study links data on children enrolled in a prospective birth cohort (Environments for Healthy Living [EFHL]: Griffith Birth Cohort study [10], registered *Australian and New Zealand Clinical Trials Registry ACTRN12610000931077*) to data from i) the Emergency Department Information System (EDIS) of four public hospitals, ii) the nationwide Medicare Benefit Scheme (MBS) and iii) the Pharmaceutical Benefits Scheme (PBS).

### Setting

From 2006 to 2011 inclusive, pregnant women from 24 weeks gestation who attended one of the only three public hospitals in the area with a birthing service were enrolled in the EFHL cohort [10]. This area services a population of approximately 800,000 people. A fourth new public hospital ED opened in the area in September 2007. ED data were available for EFHL children for the period from November 2006 to December 2013.

### EFHL data

The EFHL dataset included maternal, pregnancy/child, and household data and was collected by self-completed questionnaires by the primary caregiver at enrolment and when their child reached 12 months, 3 and 5 years of age. The baseline survey consisted of 48 self-report items including maternal, household and demographic factors during pregnancy [11]. Parents also provided consent at the time of enrolment to access additional gestational and birth information from hospital perinatal records after the delivery, hospital data and emergency department data.

### Study subjects, outcome definition and comparison groups

**Fig. 1:**
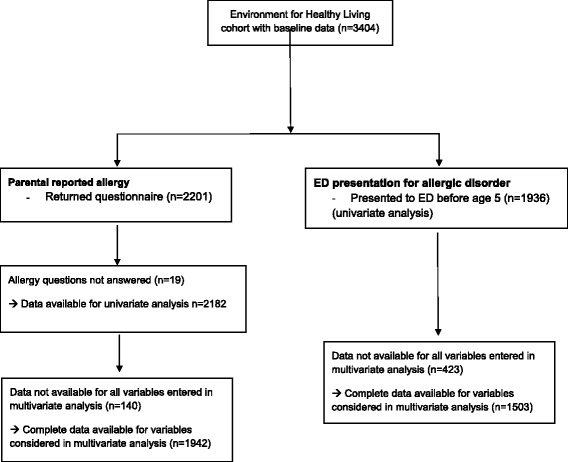
ICD-10 codes included in the definition of allergy

Study subjects were children born to mothers enrolled in the EFHL study. The primary outcomes of interest included the rate of parental-reported allergic disorders as obtained through questionnaires returned at 1, 3 and 5 years (Table 1), and the rate of ED presentations with an ICD-10 code of allergy or allergic disorder (Fig. 1). Asthma was not included as an allergic disorder. We included ‘rash’, as an allergic disorder, although other etiologies could be the cause of this diagnosis. As such (sensitivity) analyses were conducted for ED diagnoses of allergy with and without ‘rash’ included. Mothers or primary caregivers completed the surveys and are hereafter grouped as *parents* for ease of reporting. The one-year questionnaire data was included as source data for potential risk factors (e.g. breast feeding or introduction of solids) for the parental-report of allergy analysis, comparing risk factors amongst children with and without a parental-reported allergy. For the ED presentation analysis, baseline questionnaire data were used to identify risk factors for children with an ED presentation for allergy, compared to children with other ED presentations. For both parental-report and ED presentation analyses, PBS data were utilized. Table 2 summarises the available subjects for both parental-reported allergy analysis and ED presentation analysis.

**Table 1:** Enrolment into the EFHL cohort study, questionnaire response rates, and consent to Pharmaceutical Benefits Scheme (PBS) linkage

Cohort year	2006	2007	2008	2009	2010	2011	Total
Live births	*n* = 631	*n* = 477	*n* = 456	*n* = 628	*n* = 715	*n* = 497	*n* = 3404
Questionnaire returned at:							
12 months	507 (80.3%)	354 (74.2%)	308 (67.5%)	404 (64.3%)	398 (55.8%)	230 (46.3%)	2201 (64.7%)
3 years	391 (62.0%)	279 (58.5%)	230 (50.4%)	317 (50.5%)	348 (48.8%)		1565 (46.0%)
5 years	271 (42.9%)	196 (41.1%)	181 (39.7%)				648 (19.0%)
Consent given to PBS linkage							
	352 (55.8%)	272 (57.0%)	292 (64.0%)	385 (61.3%)	391 (54.8%)	220 (44.3%)	1912 (56.2%)

*EFHL* Environments for Healthy Living

**Table 2:** Samples used for analysis of i) self-reported allergy and ii) ED presentation with allergic disorder

ICD-10	Description
J30.1	Hay fever or allergic rhinitis due to pollen
J30.4	Allergic rhinitis, unspecified
J45.0	Allergic rhinitis with asthma or predominantly allergic asthma
J67^a^	Allergic alveolitis and pneumonitis due to inhaled organic dust and particles of fungal, actinomycetic or other origin
K52.2^a^	Allergic gastroenteritis and colitis
L20.0^a^	Atopic dermatitis
L20.8^a^	Atopic dermatitis
L20.9	Atopic dermatitis
L23.0	Allergic contact dermatitis due to metals
L23.9	Allergic contact dermatitis unspecified
L50.0	Allergic urticaria
L50.9	Urticaria, unspecified
R21	Rash and other nonspecific eruptions
T78.0^a^	Anaphylactic shock due to adverse food reaction
T78.2	Anaphylactic shock, unspecified
T78.4	Allergy, NOS
T80.5^a^	Anaphylactic shock due to serum
T88.1	Rash following immunization
T88.6^a^	Anaphylactic shock due to medication properly administered
T88.7	Allergic reaction to medicine properly administered

^a^Although these ICD-10 codes were eligible for inclusion, there were no cases of children in this study with these codes. ICD-10: International Classification of Disease (tenth edition)

### Administrative data sources

Table 1 summarizes enrolment and consent timeframes for relevant data sets in this study. The following administrative databases were linked to the EFHL data:

#### EDIS data

Routinely collected state-wide data from EDIS were extracted from the four public hospitals. This included baseline variables (such as hospital name), ED process variables (i.e. triage category, discharge destination) and clinical variables (i.e. primary presenting complaint and ICD-10 diagnostic codes). The triage scale used in Australia is the Australasian Triage Scale (ATS), a five-tiered scale that categorizes presentations by urgency, from 1 (immediate review and treatment required) to 5 (treatment/review required within 120 min) [12].

Linkage between EFHL participants and EDIS datasets used a unique identifier and was completed by personnel at the Health Statistics Branch of the Queensland Department of Health, and by the Health Economics and Casemix Unit, Northern NSW Local Health District.

#### MBS and PBS data

Over half of parents (56%) provided consent to access the Australian government databases of MBS and PBS. Linkage between EFHL and PBS was enabled using participant Medicare numbers. Linkage to our unique study identifier (ChildID), including manual matching and cleaning of conflicts was completed by Medicare Australia. Only prescriptions supplied prior to presentation date or follow-up time point (1 yr., 3 yr., 5 yr) were counted when comparing medication usage between children with and without allergy.

### Statistical analysis

EFHL data was managed with Stata 12.1. Data analyses were undertaken using *R* [13]*.* Chi-square tests were used to compare associations between parental-reported allergy and potential risk factors. The presentation rate to ED of children with allergic disorders was calculated based on the whole EFHL cohort (*n* = 3404, Table 1). A *p*-value of < 0.05 was considered statistically significant. Prevalence rates and 95% confidence intervals were computed using the Mid-p exact test for person-time rates. Person-years (PYs) for prevalence rates of parental-report allergy were calculated by adding the number of years each child had contributed to the study. Person-years for the prevalence of presentation to ED with allergy was calculated for each child based on the last date of ED data available (31 December 2013) minus the child’s date of birth, and summed across the cohort. Logistic regression analyses were conducted to identify variables independently associated with parental report of allergy as well as ED diagnosis of allergy (including and excluding ‘rash’). For the parental report model, variables significant at a *p* < 0.10 level in the univariate analysis and with complete data on at least 90% of children were considered in a forward stepwise conditional regression model with entry and exit criteria of *p* < 0.10. The same regression technique was used for models determining variables associated with ED presentation for allergy (including and excluding “rash” lead to two separate models). However, because some of the key univariate predictors had > 10% missing data for this model, various models were built on the full sample as well as for subsets with complete data on key variables found to be significant in univariate analysis. Interactions between variables were assessed; significant interactions (*p* < .05) were accounted for in all models. In determining the best model, the number of records, the strength of the associations, the persistence of covariates across models, and the Negelkerke’s R square value were considered.

### Ethics approval

The Human Research Ethics Committees of both the participating health service districts, and Griffith University approved this study, including linkage of data. For each participant written informed consent was obtained (from the parent or primary caregiver) for completion of a maternal baseline survey, the release of hospital perinatal data related to the birth of their child and linkage of their child’s inpatient state hospital records.

## Results

### Parental-report of allergy in children

Of the 3404 children in EFHL, questionnaire data for at least one time point were available for 2452 unique children (72%), including 2201 children with available one-year data (Table 1). The allergy questions were answered for 2182 children at 1 year, and for 1213 and 627 children at 3 and 5 years, respectively (Table 1). Allergy at any time in the child’s life was reported in 7.8% of children at 1 year, in 7.8% of children at 3 years, and in 12.6% of children with 5 years follow-up (Table 3). By 5 years of age, 255 children had an allergy as reported by their parent, representing 10.4% of the starting cohort of children who returned at least one questionnaire.

**Table 3:** Proportion of children with a parental-report of allergy by age attained, and cumulative prevalence rate of children with parental report of allergy per 1000 person-years

	12 months		3 years		5 years	
	*n* = 2182		*n* = 1213		*n* = 627	
	Person-years =2182		Person-years =4608		Person-years =5862	
	*n*	(%)	*n*	%	*n*	%
Parental reported allergy						
Yes	171	7.8%	93	7.8%	56	12.6%
No	2011	92.2%	1115	92.3%	388	87.4%
Cumulative number of unique children with allergy	171		227		255	
Cumulative prevalence rate per 1000 person-years [95% CI]	78.4 [67.3, 90.8]		49.3 [43.2, 56.0]		43.5 [38.4, 49.1]	

*CI* confidence interval

Table 4 describes univariate analysis of parental report of allergy by duration of follow up and potential risk factors in proportions and person-years, respectively.

**Table 4:** Parental-report of allergy by child’s age at follow-up and for all ages combined, and potential risk factors

	Age at follow-up											
Characteristic	12 months (*n* = 2182)			3 years (*n* = 1213)			5 years (*n* = 627)			All ages combined		
	Total N	n with allergy	% with allergy	Total N	n with allergy	% with allergy	Total N	n with allergy	% with allergy	Total person-years	Rate ratio	RR [95% CI]
Gender												
Male	1094	93	8.5%	571	56	9.8%*	211	36	17.1%**	2658	69.6	1.4 [1.2,1.8]
Female	1028	74	7.2%	607	37	6.1%	226	19	8.4%	2693	48.3	1.0 (Reference)
Maternal Indigenous status												
Not indigenous	2065	159	7.7%	1177	93	7.9%	433	55	12.7%	5286	58.1	1.0 (Reference)
ATSI	29	4	13.8%	0	0	0.0%	0	0	0.0%	29	138.0	1.8 [0.6, 4.7]
Birthweight												
< 2500 g	59	7	11.8%	33	3	9.1%	0	0	0.0%	125	79.8	1.3 [0.7, 2.5]
≥ 2500 g	1905	160	8.4%	1154	90	7.8%	426	55	12.9%	4212	59.3	1.0 (Reference)
Season of birth												
Summer/Spring	1873	148	7.9%	958	68	7.1%	389	44	11.3%	4568	56.9	1.0 (Reference)
Autumn/Winter	301	22	7.3%	240	25	10.4%	56	12	21.4%	894	66.0	1.2 [0.8, 1.5]
Maternal education												
Did not complete high school	312	29	9.3%	186	21	11.3%	64	7	10.9%	812	70.2	1.2 [0.9, 1.6]
Completed high school	1679	141	8.4%	1000	72	7.2%	380	49	12.9%	4438	59.0	1.0 (Reference)
Household income (annual)												
< $40,000	347	26	7.5%	175	11	6.3%	60	11	18.3%	816	58.8	0.8 [0.7, 1.1]
$40,000 - $70,000	635	47	7.4%	357	25	7.0%	144	15	10.4%	1638	53.1	
≥ $70,000	906	77	8.5%	511	47	9.2%	188	24	12.8%	2303	64.3	1.0 (Reference)
Other children living in household												
0	973	72	7.4%	492	32	6.5%	176	25	14.2%	2310	55.9	1.0 (Reference: 0–2)
1–2	734	47	6.4%	418	33	7.9%	167	18	10.8%	1903	51.5	
3 or more	532	50	9.4%	276	27	9.8%	95	13	13.7%	1273	70.7	1.3 [1.0, 1.7]
Child care attendance (in first year of life)												
Yes	571	52	9.1%	816	62	7.6%		Not applicable		2203	51.7	1.0 (Reference)
No	1575	115	7.3%	380	30	7.9%				2335	62.1	1.2 [0.9, 1.5]
Breast feeding before discharged newborn												
Yes	1072	89	8.3%	524	33	6.3%	204	23	11.3%	2527	57.4	1.2 [0.7, 2.1]
N0	106	5	4.7%	51	5	9.8%	30	3	10.0%	268	48.4	1.0 (Reference)
Not asked	935	72	7.7%	604	55	9.1%	200	29	14.5%	2544	61.3	Not included
Ever breast-fed by 12 months of age												
Yes	2025	160	7.9%	1053	80	7.6%	408	53	13.0%	4946	59.2	1.1 [0.6, 1.9]
No	132	9	6.8%	63	5	7.9%	0	0	0.0%	259	54.1	1.0 (Reference)
Breastfeeding duration												
0–3 months	720	59	8.2%	361	26	7.2%	138	17	12.3%	1718	59.4	1.0 (Reference all < 6 months)
3–6 months	388	31	8.0%	196	9	4.6%	77	9	11.7%	933	52.5	
> 6 months	500	36	7.2%	276	27	9.8%	105	19	18.1%	1261	65.0	1.1 [0.9, 1.5]
Age at first consumption of solids												
0–3 months	286	24	8.4%	143	5	3.5%	48	5	10.4%	668	50.9	1.0 (Reference all < 6 months)
3–6 months	1759	139	7.9%	916	76	8.3%	350	42	12.0%	4291	59.9	
> 6 months	92	7	7.6%	55	5	9.1%	26	6	23.1%	254	70.9	1.2 [0.8, 1.9]
Smoking during pregnancy												
Yes	423	41	9.7%	247	19	7.7%	89	5	5.6%*	1095	59.4	1.0 [0.8, 1.3]
No	1743	129	7.4%	949	74	7.8%	355	50	14.1%	4350	58.2	1.0 (Reference)
Use of antibiotics (in first year of life)												
None	335	69	20.6%**	232	44	19%***	41	7	17.1%	880	136.4	2.3 [1.6, 3.2]^a^
1–4 prescriptions	226	21	9.3%	167	12	7.2%	47	7	14.9%	653	61.2	4.2 [2.3, 8.4]^b^
5+ prescriptions	34	4	11.7%	128	5	3.9%	7	1	13.9%	305	32.8	

*ATSI* Aboriginal or Torres Strait Islander, *CI* Confidence Interval

^a^Reference group for nil prescriptions vs. 1–4

^b^Reference group for nil prescriptions vs. 5 +

* *p* < .05; **p < .01; ***p < .001, Reference = reference group

Parents of male children were more likely to report allergy in their children at 3 and 5 years (Table 4). The cumulative prevalence of allergy for boys over the period was 1.4 times higher than that for girls (Risk Ratio [RR] 1.4 [95% CI 1.2,1.8], Table 4).

Children with three or more other children in the household had a higher risk of allergy compared to children with 0–2 other children in the household (RR = 1.3 [1.0, 1.7], Table 4).

There appeared to be a trend towards lower parental-report of allergy in children who had solids introduced in the first 3 months of life compared to other children, most notable at the 3-year and 5 year time points (Table 4).

At 5 years, parents who were non-smokers during pregnancy reported more allergic disorders in their children than parents who smoked (14.1% vs. 5.6%; *p* = 0.03, Table 4). This finding did not persist when the cumulative rate was considered across all time points (Table 4).

The following (potential risk) factors were not significantly associated with parental-reported allergy in univariate analysis: season of birth, birthweight, maternal education level, household annual income, childcare attendance, breast feeding (ever, and duration), and passive smoking exposure (Table 4).

The logistic regression model considered data on 1942 children; 235 of these had an allergy reported at any time during follow-up. Variables considered in the model were: breast feeding at 12 months, aboriginal or Torres strait island ethnicity (ATSI), birth season, mother’s age, mother’s education status, other children at home (0,1–2 or 3+), smoking during pregnancy, mother’s place of birth, passive smoke exposure, gender, birthweight (< 2500 g and > =2500 g), gestational age (< 37 weeks vs 37+ weeks), age at first food (< 3 months, 3–6 months, 6+ months), child has asthma, child has eczema, and child care (ever) (data not shown). The interaction term of parental-report eczema and parental-report asthma was significant (*p* = 0.003), with a parental-report of asthma or eczema (+/− asthma) significantly associated with parental-report allergy. These variables were thus combined in the model as one variable according to the magnitude of their effect on self-report allergy, with a coding of neither (reference), parental-report of asthma (but no eczema), and parental-report of eczema (with or without asthma).

The following variables were univariately statistically significantly associated with parental-reported allergy: birthweight (continuous), age at first food, child has asthma, child has eczema, child care (ever). When combined with other potentially predictive variables, Table 5 shows the best model identified included the following significant variables: parental-report of asthma (adjusted Odds Ratio: aOR 2.2, 95% CI 1.4–3.4), parental-report of eczema (aOR 4.3, 95% CI 3.1–6.1) whether the child had attended childcare (aOR 1.4, 95% CI 1.1–1.9), and age of first solid intake > 6 months (aOR 1.3, 95% CI 1.0–1.7).

**Table 5:** Logistic regression results: Variables significantly associated with parental report of allergy by 5 years of age in 1942 children from a birth cohort

Variable	Total children (n)	Self-report allergy (%)	Crude odds ratio (95% CI)	*P* value	Adjusted odds ratio (95% CI)	*P* value
Child care status:						
Attended childcare	1283	12.9%	1.4 (1.1, 1.8)	0.017	1.4 (1.1, 1.9)	0.019
Did not attend childcare	994	9.7%	1.0^a^		1.0^a^	
Other self-report conditions:						
Child has neither eczema nor asthma	1742	8.8%	1.0^a^		1.0^a^	<.001
Child has asthma but no eczema	176	16.5%	2.0 (1.3, 3.1)	<.001	2.2 (1.4,3.4)	
Child has eczema (+/− asthma)	248	28.6%	4.1 (3.0, 5.7)	<.000	4.3 (3.1,6.1)	
Age (months) at first food						
< 3 months	68	7.4%	NA	0.07^b^	1.0^ac^	
Between 3 and 6 months	1403	11.3%			1.0^ac^	
6 months and older	635	13.5%			1.3 (1.0, 1.7)^c^	0.05

^a^Reference category

^b^chi-square for linear trend

^c^< 3 and 3–6 months combined as reference group

### ED presentations with allergic disorders

There were a total of 5118 ED presentations in this cohort of children aged 0–5 years. Allergic disorders (not including asthma), accounted for 3.6% (182) of these presentations from 160 of the 3404 children in the cohort. The median ED length of stay was 1.9 h. Fifteen children (8.2%) were admitted to hospital; most were assigned an Australian Triage Scale (ATS) category 3 (59%) or 4 (23%), with 14% receiving a more urgent classification (ATS 2; 13% and ATS 1; 1.1%).

Over one-third of presentations (66 of 182) with allergic disorders occurred during the first year of life (Table 6). There were two presentations due to anaphylaxis yielding a prevalence of 0.59 per 1000 PYs for anaphylaxis in the first 5 years of life.

**Table 6:** Number and cumulative prevalence rate of presentations to ED with an allergic disorder, by age group

Allergic disorder - type	By 12 months, *n*	by 3 years, *n*	by 5 years, *n*
Rash	36	62	70
Allergic reaction, NOS	6	27	40
Urticaria, NOS	8	24	30
Adverse reaction to medication	4	10	11
Allergic contact dermatitis	6	11	16
Allergic rhinitis	1	5	8
Atopic dermatitis	3	3	3
Anaphylaxis	1	1	2
Other	1	2	2
Total presentations, n	66	145	182
Total person-years	3404	9871	14,023
Prevalence per 1000 person-years [95%CI]	19.4 [15.1, 24.5]	14.7 [12.4, 17.2]	13.0 [11.2, 15.0]

*ED* Emergency Department, *CI* Confidence Interval, *NOS* Not otherwise specified

By 12 months of age, 1.8%, of the cohort had presented to ED with an allergic disorder. There was a decreasing cumulative prevalence of allergy presentation to ED, from 19.4 per 1000 person-years to 13.0 per 1000 person years as the children grew older (Table 6). ED presentation with allergy by 1 year of age occurred at a quarter of the rate of parental report of allergy (19.4 per 1000 PY compared to 78.4 per 1000 PY, Tables 3 and 6).

Univariate analysis showed that children who presented to the ED with an allergy during the first 5 years of life were more likely to be born in spring and have a mother with asthma. (Table 7). These findings persisted in the multivariate analysis as shown in Table 8, with an adjusted odds ratio for ED presentation with allergy (including diagnosis of rash) of 1.7 [95% CI 1.1–2.7] and 2.3 [95% CI 1.1–4.9], respectively. The same variables were found to be independent predictors of similar magnitude if diagnoses of ‘rash’ were excluded (Table 9).

**Table 7:** Characteristics of children presenting to ED in the first five years of life: children presenting with allergy compared to all other children presenting

Characteristic	Child with allergy presentation (*n* = 160)		Child with other ED presentation (*n* = 1776)	
	*n*	%	*n*	%
Gender				
Male	83	52.9%	948	54.2%
Female	74	47.1%	801	45.8%
Maternal Indigenous status				
Not indigenous	151	98.1%	1665	97.8%
ATSI	3	1.9%	37	2.2%
Birthweight				
< 2500 g	3	1.9%	43	2.5%
≥ 2500 g	155	98.1%	1704	97.5%
Season of birth*				
Spring^a^	113	70.6%	1064	59.9%
Summer	13	8.1%	267	15.0%
Autumn	4	2.5%	56	3.2%
Winter	30	18.8%	389	21.9%
Maternal education				
Did not complete high school	41	25.6%	376	21.2%
Completed high school	119	74.4%	1400	78.8%
Household income (annual)				
< $40,000	38	27.9%	335	22.7%
$40,000 - $70,000	40	29.4%	509	34.5%
> $70,000	58	42.6%	632	42.8%
Mother’s country of birth				
Australia/ New Zealand	99	61.9%	1069	60.2%
Other	61	38.1%	707	39.8%
Other children living in household				
0	37	35.9%	463	41.4%
1–2	59	57.3%	564	50.4%
3 or more	7	6.8%	91	8.1%
Mother has asthma*				
Yes^b^	9	8.0%	53	3.7%
No	103	92.0%	1365	96.3%
Child care attendance by 1 yr				
Yes	30	27.3%	318	27.0%
No	80	72.7%	861	73.0%
Child care attendance by 3 yrs				
Yes	43	61.4%	590	69.7%
No	27	38.6%	257	30.3%
Breast feeding before discharged newborn				
Yes	87	54.4%	1022	57.5%
No	9	5.6%	117	6.6%
Not asked	64	40.0%	637	35.9%
Breast feeding duration				
0–3 months	16	25.0%	213	27.8%
3–6 months	16	25.0%	185	24.1%
> 6 months	32	50.0%	369	48.1%
Age at first consumption of solids				
0–3 months	3	2.9%	47	4.1%
3–6 months	70	68.6%	754	65.9%
> 6 months	29	28.4%	344	30.0%
Smoking during pregnancy				
Yes	44	27.8%	460	26.0%
No	114	72.2%	1311	74.0%
Epi-pen prescribed				
none	159	99.4%	1768	99.5%
1+ prescriptions	1	0.6%	8	0.5%
Use of corticosteroids				
none	143	89.4%	1586	89.3%
1+ prescriptions	17	10.6%	190	10.7%
Use of antibiotics				
none	149	92.6%	1613	89.9%
1+ prescriptions	11	7.4%	163	10.1%

*ED* Emergency Department, *ATSI* Aboriginal or Torres Strait Islander

^a^RR (95% CI) for spring compared to all other seasons = 1.6 (1.1–2.2)

^b^RR (95% CI) for mother with asthma = 2.1 (1.1, 3.9)

*p < 0.05

**Table 8:** Logistic regression results: Variables significantly associated with ED presentation with allergy (including rash) vs any other condition, by 5 years of age

Variable	Total children (n)	Self-report allergy (%)	Crude odds ratio (95% CI)	*P* value	Adjusted odds ratio (95% CI)	*P* value
Season of birth						
Spring	1177	9.6%	1.6 (1.1, 2.3)	0.008	1.7 (1.1, 2.7)	0.011
Other season	759	6.2%	1.0†		1.0^a^	
Mother has asthma						
Yes	62	14.5%	2.3 (1.1,4.7)	0.026	2.3 (1.1,4.9)	0.025
No	1468	7.0%	1.0†		1.0†	

^a^Reference category

**Table 9:** Logistic regression results. Variables significantly associated with ED presentation with allergy (excluding rash) vs any other condition, by 5 years of age

Variable	Total children (*n*)	Self-report allergy (%)	Crude odds ratio (95% CI)	*P* value	Adjusted odds ratio (95% CI)	*P* value
Season of birth						
Spring	1131	5.9%	1.7 (1.1, 2.7)	0.02	2.2 (1.2, 4.0)	0.011
Other season	738	3.5%	1.0^a^		1.0^a^	
Mother has asthma						
Yes	59	10.2%	2.9 (1.2,6.9)	0.015	3.0 (1.2,7.3)	0.016
No	1419	3.8%	1.0^a^		1.0^a^	

^a^Reference category

There were no statistically significant differences in gender, mother’s socioeconomic status, the number of children living in the household, breastfeeding duration, or the age at introduction of solids for children presenting with an allergy compared to other ED presentations (Table 7).

## Discussion

This study used prospectively collected antenatal, perinatal and follow-up data from an existing birth cohort to study allergic disorders children under the age of 5 years, including their presentations to ED.

Our study was consistent with the existing literature for several other known risk factors for allergy such as male gender [14], birth in spring [15], co-existent eczema and asthma as well as timing of introduction of solids. Consistent with others we also found no association with breast feeding, parental education or household income [16].

The introduction of solids or potentially allergenic foods has received increased attention recently. While earlier recommendations suggested delayed introduction or avoidance of dairy products, fish and nuts in high-risk infants [17, 18], two recent randomised controlled studies have provided convincing data that early introduction does not cause allergy and may even be protective [19, 20]. Our study was consistent with these latter studies, suggesting an increased risk of allergy with later commencement of solids (Table 5). By virtue of the design of our study, we cannot exclude that this association of delayed solid introduction and allergy could be an example of reverse causation, where families at higher risk introduced solid foods later.

The “Hygiene Hypothesis” [21] proposes that increased incidence in allergies are linked to reduced exposure to microorganisms. Exposure to other children [16, 22] as well as attending day-care [23], have been associated with decrease in allergic disorders. Our study considered these potential exposures, but did not find clear support for this hypothesis in univariate and multivariate modelling.

Parental-report of child allergies occurred at 4 times the level of ED presentation. This is likely explained by the chronic or recurrent nature of certain allergic disorders, such as eczema or atopic dermatitis, which may lead to parents to seek medical attention in the setting of a primary care physician (GP) or outpatient paediatrician, or possibly not seek care at all, rather than attend an ED.

### Limitations

Not all parents consented to linkage with the PBS database and loss to follow-up occurred. As a result, the study may have been underpowered to find significant associations for known risk factors, although most point estimates findings were consistent with the existing literature. Also, due to the loss to follow-up, selection bias may have been introduced. Nevertheless, we have no reason to believe children with allergic disorders would have a different rate of loss to follow-up than others. We excluded asthma from our analysis, since our focus was on children between 0 and 5 years where diagnosis of asthma is challenging, due to their inability to provide reliable spirometry and the host of competing diagnoses such as bronchiolitis and reactive airway disease [24]*.* We acknowledge including asthma may have lead to different findings. ED diagnosis of allergy included patients with a diagnosis of ‘rash’, which accounted for half of the ED presentations in the first year of life for allergy and 40% of all presentations. We did not have approval to access patients’ individual medical record and are unable to comment on the exact etiology. A separate audit suggested more than half of these children have an allergic etiology. We conducted a sensitivity analysis by conducting logistic regression with and without patients with ‘rash’ and found a consistent result. As such we have decided to keep patients with rash in our descriptive analyses. We used ICD-10 coding for ED diagnosis which may have led to misclassification. For example, we noted very few cases of anaphylaxis, although our estimated incidence falls within previously reported ranges [25]. Furthermore, we cannot comment on the accuracy of parental report of allergy. Parental-report is considered a valid measurement for allergy, especially as a follow up measurement for a large cohort where patients are not routinely reviewed by a clinician [26].

Parental-report of allergy was unable to be further subdivided to examine specific drug or food associations. We had access to a detailed baseline database, but not all relevant possible predictors may have been included. Lastly, despite having access to multiple datasets, data entry and linkage may have been incomplete.

## Conclusion

In this birth cohort from southeast Queensland, more than 10% of children in the frist 5 years of life had an allergic disorder reported, with 3.6% of the cohort presenting to an ED with an allergic disorder. Parental report of eczema and/or asthma as well as introduction of solids after 6 months of age were significantly associated with parental report of allergy. Spring birth and a mother with asthma were independent predictors for an ED presentation for allergy.

## Additional file

Additional file 1: Extension of background literature in allergy - Factors associated with allergic disorders. (DOC 178 kb)

## Abbreviations

ATS: Australian Triage Scale
CI: Confidence Interval
ED: Emergency Department
EDIS: Emergency Department Information System
EFHL: Environments for Healthy Living
GP: General Practitioner (Family Doctor)
ICD-10: International Classification of Disease – tenth edition
MBS: Medicare Benefit Scheme
NSW: New South Wales
PBS: Pharmaceutical Benefit Scheme
PY: Person year
RR: Relative Risk
