# Supplementary material for: Parental-reported allergic disorders and emergency department presentations for allergy in the first five years of life; a longitudinal birth cohort
Source: BMC Pediatr. 2018 May 22;18:169. doi: 10.1186/s12887-018-1148-1 (PMC5964731; doi:10.1186/s12887-018-1148-1)
Supplement: Supplementary file 1 — Extension of background literature in allergy - Factors associated with allergic disorders. (DOC 178 kb) [file 12887_2018_1148_MOESM1_ESM.doc]

***Appendix 1 – Factors associated with allergic disorders***

*Factors associated with allergic disorders - Undisputed risk factors*

An undisputed characteristic associated with increased risk of any allergy is a family history of atopy ^1-4^. For development of a food allergy (FA), it is accepted that male children have a higher risk. Of interest, male gender is no longer a risk factor for food allergy in adulthood ^1, 2, 5^. For atopic dermatitis (AD), the sexes are at equal risk until age 6, and subsequently, AD occurs more often in females compared to males ^4^,

*Factors associated with allergic disorders – mildly protective (RR 0.5-0.9)*

The development of FA and other allergies in childhood can be prevented by preventing AD ^4, 6^, This phenomenon whereby AD creates a vulnerability to other allergy is called the “atopic march”^4^, and is supported by two recent RCTs showing moderate reduction in the development of AD (odds ratios between 0.25 and 0.49) if moisturizer was regularly applied during infancy ^7, 8^. Not only was there a reduced incidence of AD in infants in the regular moisturizer arm, but these infants also had fewer incidences of FA and allergic rhinitis.

The hygiene hypothesis, or microbiome hypothesis, asserts that a reduction in microbial exposure has caused the increase in allergy prevalence. Recent studies examining faecal samples in infants confirm that there are links between the composition of the flora in the microbial gut and development of AD ^9^. However, the particular bacteria of interest have not been identified. It also appears that the composition of the microbial gut is affected by a variety of factors under ongoing investigation as causal factors for allergy, including antibiotic use, vitamin D, probiotic use, breast feeding, pets in the household, and number of siblings ^9, 10^. As such, some hygiene-related exposures that most experts agree are ***mildly*** protective against allergy development are summarized in Table 1. Some dietary exposures are also thought to reduce risk of FA development in childhood to a small (but statistically significant) extent (Table 1).

There is general consensus amongst experts there is no consistent association identified between the following, potentially causative or protective, factors for atopy or FA development. The published evidence is of sub-optimal quality, and the direction and strength of the relationships of the factors of interest is variable. Some previously believed protective factors have now been shown to have no significant association, see Table 2.

Table 1. Factors associated with allergic disorders – considered mildly protective

|  | **Hygiene Hypothesis – reducing risk of allergy** | **Dietary exposure – reducing risk of allergy** |
| --- | --- | --- |
|  | Pet exposure prenatally ^9^ and during the first year of life (ORs 0.6 to 0.8) ^1, 2, 9, 11^, or exposure to farm animals ^12^ | Early introduction of solid food during infancy (after 17 weeks of age) is protective against development of FA ^1, 2^, and recommended, including potentially allergenic foods ^3, 13-16^ |
|  | Older siblings in the home for food allergy ^1, 2^, or family size for AD^4^ | Use of hydrolysed infant formula for infants that cannot be exclusively breast-fed, reduces risk of AD mildly in infants with a family history of atopy ^13, 15, 16^ |
|  | Day care attendance ^12^ | Breast feeding protects against AD in infants with a family history of atopy^16^ |
|  | Probiotics for AD ^2, 11^, and particularly if administered in late pregnancy and early infancy ^17^; Some experts, however, are not ready to endorse use of probiotics ^6, 15^ | Increased food diversity in infancy reduces FA risk ^1^  Exposure to peanuts after 4 months of age reduces peanut allergy risk ^18^ |
|  | Diversity in microbial exposure during infancy^12^ | Maternal supplementation with fish oil during pregnancy reduces allergy development ^17^ |
|  | Probiotics in infancy ^10^ | Maternal diet high in potentially allergenic foods is protective^13^, as is maternal diet low in vegetable oil, margarine and processed foods during pregnancy and lactation ^17^ |

AD: atopic dermatitis, FA: food allergy

Table 2. Factors not (consistently) associated with allergic disorders

|  | ***Variable direction and strength of evidence*** | ***Previous associations now ‘debunked’*** |
| --- | --- | --- |
|  | Caesarean section vs. vaginal birth ^9, 19^ | Maternal avoidance of potentially allergenic foods during pregnancy and breast feeding (^2, 11, 15-17^) |
|  | Vitamin D exposure in utero or during early infancy ^1, 2, 6, 9-11, 17^ | Avoidance of potentially allergenic foods during infancy (after 4-6 months of age) ^2, 15, 17^ |
|  | Breast feeding in general ^3, 9, 11, 17^ |  |
|  | Antibiotic use in pregnancy* ^9^, or during early infancy ^19, 20^ |  |
|  | Lower socioeconomic status and lower maternal education ^4^ |  |

**References Appendix 1**

[1] Savage J, Johns CB. Food allergy: epidemiology and natural history. Immunology and allergy clinics of North America. 2015;35:45-59.

[2] Sicherer SH, Sampson HA. Food allergy: Epidemiology, pathogenesis, diagnosis, and treatment. The Journal of allergy and clinical immunology. 2014;133:291-307; quiz 8.

[3] Longo G, Berti I, Burks AW, Krauss B, Barbi E. IgE-mediated food allergy in children. Lancet (London, England). 2013.

[4] DaVeiga SP. Epidemiology of atopic dermatitis: a review. Allergy Asthma Proc. 2012;33:227-34.

[5] Lack G. Update on risk factors for food allergy. The Journal of allergy and clinical immunology. 2012;129:1187-97.

[6] Cipriani F, Dondi A, Ricci G. Recent advances in epidemiology and prevention of atopic eczema. Pediatric Allergy and Immunology. 2014;25:630-8.

[7] Horimukai K, Morita K, Narita M, Kondo M, Kitazawa H, Nozaki M, et al. Application of moisturizer to neonates prevents development of atopic dermatitis. The Journal of allergy and clinical immunology. 2014;134:824-30 e6.

[8] Simpson EL, Chalmers JR, Hanifin JM, Thomas KS, Cork MJ, McLean WH, et al. Emollient enhancement of the skin barrier from birth offers effective atopic dermatitis prevention. The Journal of allergy and clinical immunology. 2014;134:818-23.

[9] Wegienka G, Zoratti E, Johnson CC. The Role of the Early-Life Environment in the Development of Allergic Disease. Immunology and Allergy Clinics of North America. 2015;35:1-17.

[10] Campbell DE, Boyle RJ, Thornton CA, Prescott SL. Mechanisms of allergic disease–environmental and genetic determinants for the development of allergy. Clinical & Experimental Allergy. 2015;45:844-58.

[11] Madhok V, Futamura M, Thomas K, Barbarot S. What's new in atopic eczema? An analysis of systematic reviews published in 2012 and 2013. Part 2. Treatment and prevention. Clinical and experimental dermatology. 2015;40:349-55.

[12] Brooks C, Pearce N, Douwes J. The hygiene hypothesis in allergy and asthma: an update. Curr Opin Allergy Clin Immunol. 2013;13:70-7.

[13] Sampson HA, Aceves S, Bock SA, James J, Jones S, Lang D, et al. Food allergy: a practice parameter update—2014. Journal of Allergy and Clinical Immunology. 2014;134:1016-25. e43.

[14] Heinrich J, Koletzko B, Koletzko S. Timing and diversity of complementary food introduction for prevention of allergic diseases. How early and how much? Expert review of clinical immunology. 2014;10:701-4.

[15] Chalmers JR, Bremmer SF, Simpson EL. The Primary Prevention of Atopic Dermatitis. Evidence-Based Dermatology: John Wiley & Sons, Ltd; 2014. p. 127-35.

[16] Fleischer DM, Spergel JM, Assa'ad AH, Pongracic JA. Primary Prevention of Allergic Disease Through Nutritional Interventions. The Journal of Allergy and Clinical Immunology: In Practice. 2013;1:29-36.

[17] Julia V, Macia L, Dombrowicz D. The impact of diet on asthma and allergic diseases. Nat Rev Immunol. 2015;15:308-22.

[18] Du Toit G, Roberts G, Sayre PH, Bahnson HT, Radulovic S, Santos AF, et al. Randomized trial of peanut consumption in infants at risk for peanut allergy. N Engl J Med. 2015;372:803-13.

[19] Madhok V, Futamura M, Thomas K, Barbarot S. What's new in atopic eczema? An analysis of systematic reviews published in 2012 and 2013. Part 1. Epidemiology, mechanisms of disease and methodological issues. Clinical and experimental dermatology. 2015;40:238-42.

[20] Kuo CH, Kuo HF, Huang CH, Yang SN, Lee MS, Hung CH. Early life exposure to antibiotics and the risk of childhood allergic diseases: An update from the perspective of the hygiene hypothesis. J Microbiol Immunol Infect. 2013;46.
